# Ophthalmology

**Published:** 1908-09

**Authors:** Cyril H. Walker


### OPHTHALMOLOGY.

The extensive use of protargol, argyrol, and other silver-albumen preparations in cases of **ophthalmia neonatorum** has of late been somewhat checked by the publication of the results of laboratory experiments. For example, the admirable work of Kelly[1] shows that to destroy staphylococcus pyogenes aureus, the

[1] *Brit. M. J.*, 1907, ii. 1475.

solution of protargol has to be 256 times as strong as a silver nitrate solution in order to produce the same effect. The bactericidal action of argyrol is even less than that of protargol, and many have therefore assumed that it has little or no clinical value. In consequence of this, many practitioners are reverting to the use of nitrate of silver solutions.

Cragin, in the *American Journal of Obstetrics*, July, 1907, publishes the results of prophylactic methods in 8,000 confinements at the Sloane Maternity Hospital. In ophthalmia neonatorum he includes all cases of purulent conjunctivitis in the new-born. The latest statistics show that 67.14 per cent. of these cases are due to infection with gonococci. Of the remainder, about 10 per cent. are due to pneumococci, 8 per cent. to bacterium coli, whilst a smaller proportion are due to Koch-Weeks' bacillus, streptococci, staphylococci, &c., acting either separately or in association. Owing to the enormous preponderance of gonococcal cases (which include all the more serious ones), distinction between the various causative agents is hardly necessary. The following silver solutions were used, viz. silver nitrate, 2 per cent. (Credé's method) ; silver nitrate, 1 per cent. ; protargol, 5 per cent..; argyrol, 10 and 20 per cent. The proportion of cases infected, in spite of the prophylactic used, varied from 18 per thousand with the 2 per cent. silver nitrate to 34 per thousand with the 1 per cent. silver nitrate. The 4,000 cases treated with the argyrol solutions showed about as good results as the silver nitrate cases. During the use of the 2 per cent. nitrate of silver solutions, the irritation of the eyes, with the accompanying œdema and discharge, was so great that it seemed to be a source of danger, although no eyes were lost, and no corneal opacities produced. He therefore used a 1 per cent. solution for the second thousand cases. Testing the bactericidal powers of silver salts, Cragin found that argyrol had practically no effect on streptococcus and staphylococcus, but that a 20 per cent. was rapidly fatal to the gonococcus. Assuming that the gonococcus is the microbe to be most dreaded, and that argyrol gives as good clinical results as 2 per cent. silver nitrate (and better results than the 1 per cent. solution), he advocates the use of argyrol as the best prophylactic against ophthalmia neonatorum. For the curative treatment he recommends cleansing with boracic lotion every twenty minutes, and the instillation of 20 or 30 per cent. argyrol every two to four hours. In cases of ophthalmia which resist argyrol, he advises the occasional use of 1 or 2 per cent. nitrate of silver solutions.

In his recent work on *Ophthalmia Neonatorum*, Sydney Stephenson to a great extent confirms the above statements. He, however, prefers a 1 per cent. nitrate of silver solution as a prophylactic, which, he says, "is fully protective, and in the present state of our knowledge may be regarded as the most efficient solution to employ in the preventive treatment of ophthalmia neonatorum." Statistics collected by various investigators of more than 36,000 babies show less than five cases per thousand when this prophylactic is used. A 1 per cent. solution of nitrate of silver would appear to have certain practical advantages: it is cheap, and may be fairly safely used by an imperfectly instructed midwife. It is usually easy to tell if the solution has been spoilt by keeping. Protargol and argyrol are relatively much more expensive, solutions are more difficult to prepare, and do not keep well. Some of the disappointing results from their use are no doubt due to this last fact.

In the treatment of ophthalmia neonatorum, Stephenson fully agrees with Cragin as to the great value of argyrol, but would not recommend its use more than three or four times in the twenty-four hours.

**Recurrent paralysis of the third nerve.**—Prof. C. E. Finlay reports a case,[1] and refers to eighty-eight other recorded cases. The condition has been known for nearly fifty years. It is most frequent in young adults, and is probably far more common (in a mild degree at any rate) than the literature of the disease would indicate. Charcot, in 1890, called it ophthalmic migraine, under which title it is still often described. Finlay recognises four stages of the affection:—1. Periodic hemicrania, with no visual aura (scotoma scintillans and hemianopsia fugax), the attacks lasting from a few hours to a few days. In this stage there is no paresis during or after the attack. 2. The attacks of pain cease suddenly, and are followed by symptoms of paralysis, generally affecting only the third nerve, but the fourth or sixth nerves may rarely be affected instead. During the intervals between the attacks the affected muscles recover. 3. Similar attacks, but paresis of one or more muscles persisting between the attacks. 4. In this stage the paralysis is permanent, the attacks of pain recurring at irregular intervals. Vision, apart from accommodation and fixation, is not affected.

The attack proper has two periods—(*a*) Sudden increasing pain centred round the eye, accompanied by nausea, lasting hours or days, and culminating in vomiting; (*b*) the period of paralysis, usually complete and total, lasting from several days to several months, followed by gradual recovery.

The symptoms must be distinguished from those found in some cases of ocular paralysis in locomotor ataxy, cerebral tumours, cerebral syphilis, nuclear paralysis, and hysterical paralysis. It is perhaps with this last that it is most likely to be confused, but the visual fields show no limitation. It has been pointed out that excepting the attacks and the paralysis, there are no nervous symptoms whatever. By a process of exclusion, the disease can be located in the basilar portion of the third nerve. Charcot regarded it as a vasomotor disturbance followed by

[1] *Arch. Ophthal.*, 1908, xxxvii. 1.

nutritive changes. Four cases have been examined *post-mortem*. All showed fibrinous exudate surrounding the third nerve. The prognosis, so far as the ultimate result is concerned, is unfavourable. Strychnine and iodide of potassium appear to offer the best prospect of relief.

**Tests for colour vision.**—A committee of the Ophthalmological Society has been appointed to investigate the question of colour blindness, and the tests for its detection. "Holmgren's Wools," no matter how carefully used, are not sufficiently accurate in all cases; the defective candidate may be passed, and one with practically perfect colour vision refused. The spectroscope furnishes the best test. By means of a fixed and a movable spectrum, fitted with suitable shutters, and both projected upon the same screen, a subject can be required to match any isolated portion of one spectrum with a similar portion of another. Though not of the same scientific accuracy, Edridge-Green's "signal lamp," which can be easily fitted up in an ordinary consulting-room, is sufficiently accurate for all practical purposes. By means of an iris diaphragm and coloured glasses, the light can be varied in colour and intensity. It would be a matter of interest and practical importance to study the effect of fatigue, both bodily and retinal, upon colour perception.

**Transplantation of the cornea.**—The editor of the *Ophthalmic Review*, in the January number, 1907, gives an epitome of what must be regarded as by far the most successful case of keratoplasty hitherto recorded. A workman, aged 45, received extensive lime burns of both corneæ. Sixteen months later Zirm was able to transplant pieces of a healthy cornea from a boy aged 11, whose right eye was so injured as to necessitate excision. The technique was as follows: Immediately after enucleation the eye was placed in warm normal saline solution. The workman was anæsthitised, and a conjunctival bridge was dissected up below the right cornea. Then, by means of v. Hippel's trephine (probably about 5 mm. diameter and worked by a spring), a disc of cornea was removed from the enucleated eye, and an exactly similar disc was removed from the leucomatous cornea. The transparent disc was placed in the gap, and the bridge of conjunctiva was drawn over it by sutures. Another disc was removed from the enucleated eye, this time from the centre of the cornea, and placed in a corresponding trephine hole in the patient's left cornea. It was an exact fit, and was secured in the same way, with a conjunctival flap. The right eye was a failure, and had to be excised, but the graft in the left eye remained clear, and in six months the vision of the left eye was 3-20 and J.13. Zirm attributes the success to the fact that the graft was from a young and healthy cornea. It was floated into position with a minimum of handling, and the patient's cornea was superficially vascular. He suggests that in future cases he would "raw" the cornea all round the area to be trephined, and cover it with a bridge of conjunctiva dissected up from below and brought right across the future gap. This would provide vascularity right up to the graft, which would be inserted as soon as the irritation had subsided.

Arnold Knapp[1] adds one more to the lengthening list of undesirable results following the use of **Calmette's ophthalmo-reaction.** The patient was a girl aged 9, with scrofulus superficial keratitis in the right eye of two months' duration. Otherwise she was healthy, and her family history good. Treatment was unsatisfactory, and an intractable ulcer formed on the lid margin. On December 14th, 1907, one drop of a 1 per cent. tuberculin solution was instilled in the left eye, which had never been affected. There was severe reaction in six hours, and the temperature rose to 100.4° F. Under treatment the swelling of the lids abated, but the conjunctiva remained red and thickened. Ten days later corneal infiltrations appeared, arranged in three groups near the margin. New blood-vessels and infiltration appeared in the superficial layers of the corneal stroma. By January 28th they had coalesced, and the infiltration had begun to spread over the centre of the cornea. The author concludes: "This is a typical tuberculous process in the cornea, and as it came on after the use of the tuberculin solution in a previously healthy eye, a connection seems very probable. This unfortunate case demonstrates that the ophthalmo-reaction cannot be regarded as harmless." The difficulty of convincing a patient (or perhaps a jury) that interstitial keratitis, occurring under such conditions, was due to a constitutional cause demands that great caution should be exercised in using the reaction. An abstract of Schiele's work[2] shows that a reaction is almost invariable in trachoma and follicular conjunctivitis. Parkes Weber states[3] that the solution placed in healthy eyes of healthy subjects produced a positive reaction if they read all the succeeding evening.

CYRIL H. WALKER.

---

### Reviews of Books.

### BOOKS ON MEDICINE.

**Heart Disease and Thoracic Aneurysm.** By F. J. POYNTON, M.D. Pp. x., 310. London: Oxford Medical Publications. 1907.

It is difficult nowadays to write a short book on diseases of the heart, and at the same time to deal adequately with the subject. Dr. Poynton, in this manual, has attained a certain

[1] *Ibid.*, 171. [2] *Oph. Rev.*, June, 1908.
[3] *Brit. M. J.*, 1908, i. 386.

